# Targeting the HIV-1 and HBV Capsids, an EnCore

**DOI:** 10.3390/v15040896

**Published:** 2023-03-31

**Authors:** William M. McFadden, Stefan G. Sarafianos

**Affiliations:** 1 Center for ViroScience and Cure, Laboratory of Biochemical Pharmacology, Department of Pediatrics, Emory University School of Medicine, Atlanta, GA 30322, USA; 2 Children’s Healthcare of Atlanta, Atlanta, GA 30322, USA

## 1. Introduction

Not many structures are common among all viruses: only nucleic acid and a protein coat. This protein coat, also called the capsid, exists to protect the viral genome in varying capacities. In some viruses, the capsid has additional roles throughout the replication cycle critical for replication, for example, in immune evasion, host cell entry, intracellular trafficking, host factor recruitment, and much more [1]. Capsids vary in shape, size, complexity, and importance; however, the presence of a capsid (in a current or ancestral form) is a requisite to be classified as a virus [2]. Further, a viable strategy in combatting some viral infections can be disrupting the capsid, and subsequently its ability to protect the genome.

While capsids do not have enzymatic activity, disrupting the inter- and intramolecular interactions of the capsid proteins could have a negative impact on viral fitness. In the case of hepatitis B virus (HBV), capsid assembly modulators (CAMs) have been subdivided into two classes: class I CAMs, which create misassembled or damaged capsids that are unable to function, and class II CAMs, which promote the rapid assembly of cores that lack the viral genome [3]. Multiple CAMs have been evaluated in clinical trials for the treatment and cure of chronic hepatitis B.

In the case of human immunodeficiency virus type 1 (HIV-1), it has been shown that both increasing and decreasing the capsid stability suppresses infectivity [4]. In 2022, the first capsid-targeting antiretroviral, lenacapavir (brand name Sunlenca), was approved for use in highly treatment-experienced patients as an addition to current antiretroviral therapy (ART) regimens [5]. Lenacapavir is highly potent, with a 50% effective concentration (EC_50_) of 31–95 pM [6], and is a long-acting compound with a current upkeep dosing at 6-month intervals [7]. Additionally, there are dozens of compounds reported to bind the HIV-1 capsid with over five mechanisms of action that stabilize or destabilize it, as well as some that interfere with host factor interactions [8,9,10]. Since the capsid relies on a highly sensitive network of interactions, the viral capsid protein is great for drug design, but it is challenging to study.

In this Special Issue of *Viruses* “Capsid-Targeting Antivirals and Host Factors”, two major motifs emerge in studies of both the retrovirus HIV-1 and the hepadnavirus HBV: (1) the importance of experimental conditions in capsid-based studies, and (2) examples of antiviral studies from discovery to investigating mechanisms of action for capsid-targeting antivirals.

## 2. The Importance of Experimental Conditions in Capsid-Based Studies

As stated before, many mechanisms of action for capsid-targeting antivirals interrupt the fine-tuned kinetics of protein–protein interactions in and with the capsid lattice. This, along with useful techniques in studying HIV-1 biology and antiviral design, was reviewed by Zhuang and Torbett [10]. Willbourne and Zhang described the advances of structural methods and the limitations of investigating the capsid protein at the atomic level [11].

Multiple reports in this Special Issue exemplified the importance of avoiding the introduction of biases that would interfere with the native capsid’s environment. Mamede et al. described and characterized fluorescently tagged protein systems to image and track HIV-1 infection [12]. They detailed the fitness of multiple labeled particles to identify the movement of the HIV-1 capsid protein (CA) without directly labeling it. Their strategy of tagging integrase prevents any aberrant function of the capsid protein and, further, is a modular system compatible with other fluorescent imaging systems [12]. At the cellular level, Francis et al. described how different cell systems can influence the kinetics of the capsid [13]. In a more physiologically relevant cell line of monocyte-derived macrophages with a reported decrease in the dNTP concentration, compared to the more widely used HeLa-derived TZM-bl cells, they reported less frequent early cytoplasmic uncoating and slower reverse transcription [13]. Overall, these studies showed the importance of both the virus system and the host cell in studying the kinetics of the HIV-1 capsid.

## 3. Targeting the Capsid with Antivirals

Screening techniques that characterize the stabilization or destabilization of the capsid proteins can help identify viable antivirals. A thermal shift assay (TSA) was employed to characterize compounds that modulate the thermal stability for both HIV-1 CA and the HBV core protein (Cp) in Sahani et al. and Senaweera et al., respectively [14,15]. Sahani et al. explored a library of compounds that target the PF74-binding site and characterized the antiviral activity of compound 15 (EC_50_ of 0.31 μM) and metabolic stability [14]. The PF74-binding site is currently the most clinically relevant site in CA, as this is the site of lenacapavir binding. Senaweera et al. described HBV CAMs from a virtual screen and validated the computational findings using the TSA and antiviral activity assays [15]. Two novel HBV CAM scaffolds, ZW-1841 (EC_50_ 6.6 ± 0.4 μM) and ZW-1847 (EC_50_ 3.7 ± 1.0 μM), warrant a further investigation for design in next-generation inhibitors [15].

The most agreed-upon mechanism of action for HIV-1 capsid-targeting antiretrovirals is impacting the capsid assembly and thus losing its protective and trafficking roles, as reviewed in AlBurtamani et al. They reviewed the role of CA during the early steps of HIV-1 lifecycle, from entry to integration [9]. This was complemented in a review by Engleman (2021), which outlined the influence of capsid in integration and provided perspective on how capsid-targeting antivirals could modify integration processes as a primary or secondary mechanism of action [16].

Two reports investigated the mechanism of actions for CAMs that act as Class II inhibitors, creating intact, tight HBV capsids. Pérez-Segura et al. [17] used molecular dynamics simulations to characterize the network of interactions in the Cp of HBV with the well-characterized compound AT130 [17]. AT130 (EC_50_ 0.13–2.4 μM) [18,19] caused rapid Cp assembly, and the interactions discussed can help explain the observed antiviral effects [17]. Hurwitz et al. further investigated the physiological effects of their recently reported CAM [20] GLP-26 (EC_50_ of 3 nM) [21]. Their findings showed that the compound is effective and well tolerated in both animal studies and primary human cardiomyocytes [21]. These promising antivirals showed the feasibility of CAMs for a further development and clinical applications toward eradicating hepatitis B.

Once placed under the selective pressures of antivirals, the virus can evolve resistance mutations that diminish antiviral activity. Lingappa et al. discussed the benefits of host-targeting antivirals over direct-acting agents; the replication cycle of the virus enables the selection of resistant mutations within the virus while the host proteins are unchangeable [22]. They further reviewed PAV-206 as a late-stage inhibitor of HIV-1 (EC_50_ of 34–75 nM) with a high barrier to resistance observed in cell-based passaging studies [22,23]. Thus, they hypothesized that PAV-206 is targeting a host factor [22,23]. Furthermore, in the recent CAPELLA trials of lenacapavir, multiple resistance mutations emerged in CA, with the most prevalent being the M66I mutation [7]. This was characterized by Sun et al. who described a potential mechanism of resistance due to the I66 side chain steric clashing with lenacapavir and high-energy reorganization of the isoleucine substituent [24]. The dramatic resistance observed for lenacapavir with M66I in reporter cells (>3000-fold change in EC_50_) was not as large for other compounds that bind at the same binding pocket: PF74 and ZW-1261 [24]. Overall, the clinical relevance of the M66I mutation heightens the need for additional capsid-targeting antivirals in treating HIV-1 infection.

## 4. Conclusions

HBV CAMs and HIV-1 capsid-targeting antiretrovirals can be an additional line of defense against antiviral-resistant viral strains as unique drug targets; however, the use of capsid-targeting antivirals can result in drug-resistant mutations just as with any other antiviral strategy. This underscores the importance of identifying compounds that target the viral capsid in both known and novel capacities. An investigation into viral capsids can result in methods to impair capsid–capsid interactions; moreover, when the capsid has additional roles in the viral replication cycle, methods to impair the capsid–host factor and the capsid–viral factor interactions can offer additional therapeutic targets. These interactions offer strategic opportunities to monitor the events of the viral replication cycle using approaches such as indirect imaging, which prevents exogenous dyes or tags from disrupting the potentially sensitive network of interactions in capsids. Overall, for any virus, the capsid offers opportunities for targeting the protective scaffold and potentially uncloaking viral nucleic acids, exposing them to innate immunity cellular sensors. Thus, research on viral capsids offers unique opportunities to elucidate the biology of viruses while also potentially leading to the discovery of novel therapeutic approaches.
